# Tuberculosis treatment adherence and fatality in Spain

**DOI:** 10.1186/1465-9921-10-121

**Published:** 2009-12-01

**Authors:** Joan A Caylà, Teresa Rodrigo, Juan Ruiz-Manzano, José A Caminero, Rafael Vidal, José M García, Rafael Blanquer, Martí Casals, the Working Group on Completion of Tuberculosis Treatment in Spain (Study ECUTTE)

**Affiliations:** 1 Programa Integrado de Investigación en Tuberculosis (PII TB) de la Sociedad Española de Neumología y Cirugía Torácica (SEPAR), Spain; 2 Unidad de Investigación de Tuberculosis de Barcelona, Servicio de Epidemiología de la Agencia de Salud Pública de Barcelona, Barcelona, Spain; 3 Fundación Respira de la SEPAR, Barcelona, Spain; 4 Hospital Universitario Germans Trías y Pujol de Badalona, Badalona, Spain; 5 Hospital General Universitario de Gran Canaria Dr Negrín, Canary Islands, Spain; 6 Hospital Vall D'Hebrón de Barcelona, Barcelona, Spain; 7 Hospital San Agustín, Avilés, Asturias, Spain; 8 Hospital Universitario Dr Peset de Valencia, Valencia, Spain; 9 CIBER de Epidemiología y Salud Pública (CIBERESP), Barcelona, Spain; 10 CIBER de Enfermedades Respiratorias (CIBERES), Barcelona, Spain

## Abstract

**Background:**

The adherence to long tuberculosis (TB) treatment is a key factor in TB control programs. Always some patients abandon the treatment or die. The objective of this study is to identify factors associated with defaulting from or dying during antituberculosis treatment.

**Methods:**

Prospective study of a large cohort of TB cases diagnosed during 2006-2007 by 61 members of the Spanish Society of Pneumology and Thoracic Surgery (SEPAR). Predictive factors of completion outcome (cured plus completed treatment vs. defaulters plus lost to follow-up) and fatality (died *vs. *the rest of patients) were based on logistic regression, calculating odds ratios (OR) and 95% confidence intervals (CI).

**Results:**

Of the 1490 patients included, 29.7% were foreign-born. The treatment outcomes were: cured 792 (53.2%), completed treatment 540 (36.2%), failure 2 (0.1%), transfer-out 33 (2.2%), default 27 (1.8%), death 27 (1.8%), lost to follow-up 65 (4.4%), other 4 (0.3%). Completion outcome reached 93.5% and poor adherence was associated with: being an immigrant (OR = 2.03; CI:1.06-3.88), living alone (OR = 2.35; CI:1.05-5.26), residents of confined institutions (OR = 4.79; CI:1.74-13.14), previous treatment (OR = 2.93; CI:1.44-5.98), being an injecting drug user (IDU) (OR = 9.51; CI:2.70-33.47) and treatment comprehension difficulties (OR = 2.93; CI:1.44-5.98). Case fatality was 1.8% and it was associated with the following variables: age 50 or over (OR = 10.88; CI:1.12-105.01), retired (OR = 12.26;CI:1.74-86.04), HIV-infected (OR = 9.93; CI:1.48-66.34), comprehension difficulties (OR = 4.07; CI:1.24-13.29), IDU (OR = 23.59; CI:2.46-225.99) and Directly Observed Therapy (DOT) (OR = 3.54; CI:1.07-11.77).

**Conclusion:**

Immigrants, those living alone, residents of confined institutions, patients treated previously, those with treatment comprehension difficulties, and IDU patients have poor adherence and should be targeted for DOT. To reduce fatality rates, stricter monitoring is required for patients who are retired, HIV-infected, IDU, and those with treatment comprehension difficulties.

## Introduction

Tuberculosis (TB) is an infectious disease requiring adherence to long-term treatment and the tracing of patient's contacts, thus justifying it being a notifiable disease in most countries of the world. This ancient disease continues to be an important public health problem, and for this reason the World Health Organisation (WHO) declared it to be a global emergency in 1993 [1]. In 2007 it was estimated that, worldwide, there had been 9.27 million new cases and 1.756 million deaths from TB, of which 1.37 million cases and 0.456 million deaths were among HIV-infected individuals[2]. Moreover, to these new cases one must add the millions already in existence, making it the most prevalent infectious disease[3].

The rise of immigration over the last decade in Spain has substantially altered the characteristics of TB patients. The Spanish population was 45,200,737 inhabitants in 2007, of whom 4,419,554 (9.99%) were foreign-born[4]. In Barcelona, a city with one of the highest influxes of immigrants, the percentage of foreign-born TB patients rose from 5% in 1995 to 46% in 2007 (with incidence rates among immigrants of over 100 cases per 100,000 inhabitants) [5].

The Tuberculosis and Respiratory Infections section of SEPAR (Spanish Society of Pneumology and Thoracic Surgery) has previously published a study on adherence to anti-tuberculosis treatment and on fatality, referring to a cohort of patients followed during the period 1999 to 2000[6]. The findings indicated that immigrant status and being an injecting drug user were associated with worse treatment adherence, while patients who were HIV-infected, alcoholics, or of advanced age presented higher fatality.

The aims of the present study were to analyse antituberculosis treatment adherence and fatality during standard TB treatments in patients with TB in Spain, and to identify factors associated with these events. The study will also permit changes in relation to the earlier study[6] to be assessed, and to determine whether demographic changes experienced in Spain due to the considerable rise in immigration have had any influence on adherence to tuberculosis treatment.

## Methods

A multicentric prospective study was carried out involving prospective follow-up of an extensive cohort of TB patients, provided by 61 collaborators from 53 hospitals throughout Spain. The study was promoted by the Integrated TB Research Programme of SEPAR. Patients diagnosed with TB between 1 January 2006 and 31 December 2007, aged 18 years or over, were included. Those patients with known resistances were excluded, as were those in whom initiation of standard TB treatment was not advisable, such as patients with hepatic problems. Cases were followed up according to an evaluation calendar (Table 1). An informed consent to participate in the study was elicited.

**Table 1:** Patient evaluation calendar

	Visit 1 Diagnosis	Visit 2 2 Months	Visit 3 * 6 Months
**Criteria of inclusion/exclusion**	X		

**Sociodemographic data**	X		

**Tobacco, alcohol, drug use**	X		

**Anthropometric data**	X	X	X

**Clinical history**	X		

**Diagnostic method employed**	X		

**Medication**	X	X	X

**Clinical course**		X	X

**Adherence to treatment**		X	X

**Collection of samples**	X	X	X

**Drug sensitivity test**	X		

**Treatment outcome**			X

**Informed consent**	X		

* For long treatments new visits are recommended at 9,12,18 months.

The information collected covered the following aspects: sociodemographic data, toxic habits, clinical history, diagnostic methods, drug-susceptibility, medication, clinical course, and adherence to and outcome of treatment. Data was collected through an electronic diary made available through a computerised application, accessed by each study collaborator via the SEPAR Web site using a personalised username and password.

Any patient born outside Spain was classified as an immigrant. Men consuming over 280 g of alcohol per week, and women over 168 g, were considered alcoholics. Intravenous users of illegal drugs (heroin and cocaine) were classified as intravenous drug users (IVDU). Toxicity was defined as an adverse effect that requires to change at least one drug, and treatment comprehension was defined as the perception of the treating doctors of the patient. The chest X-Rays were performed at the moment of the diagnosis and at the 2^nd ^and 6^th ^month and when necessary, and the evolution was classified by the treating doctor of the patient as improvement, stable or progression.

During these years the standard treatment for TB in Spain for new cases were: 2 months of rifampicin (R), isoniazid (H) and pirazinamide (Z) followed by 4 months of RH (2RHZ+4RH) or the same treatment plus Ethambutol (E) during the first 2 months^20 ^(2RHZE+4RH). In Spain DOTS is a priority only for patients with high risk of bad adherence (IVDU, homeless, prisoners, etc). A patient was included in the previous treatment category only if he or she had taken antituberculosis treatment over one year before the current active TB episode.

Control of questionnaire completion and the database was carried out via telephone and e-mail contacts between the field worker and study collaborators.

The following definitions were employed for treatment outcome, in accordance with European recommendations[7]:

*Cured*: when the patient has completed a full course of anti-TB therapy and a negative culture is obtained during the continuation phase (culture-positive patients) or two negative sputum smears during the continuation phase, one of which must be at the end of treatment (patients diagnosed by microscopy).

*Treatment completed*: if the course of treatment prescribed was completed but no bacteriological conversion occurred (culture-positive patients) or no smear result is available at the end of treatment (patients diagnosed by microscopy).

*Default*: If the patient interrupts the treatment for any reason for more than two months, if there is a non-completion of treatment within 9 months when the patient is placed on a six-month regimen, or if the drug intake was < 80% of the prescribed dose.

*Treatment failure*: A patient who fails to achieve bacteriological conversion within 5 months after the start of treatment or, after previous conversion, becomes sputum smear or culture positive again.

*Death*: A patient who died of any cause during the course of treatment is recorded under death.

In the present study, the category of transfer out[7] was redefined into two subcategories:

*Lost to follow-up*: when it is known that the patient disappeared and no additional information is available.

*Transfer out*: when a patient moves to another town or health centre and whose follow-up (with medical report available) is the responsibility of a doctor not collaborating in the present study.

The results of the analysis were summarized as:

*Successful outcome*: the percentage of patients who were cured or completed treatment out of all those detected.

*Completion outcome*: the percentage of patients who were cured or completed treatment out of all patients who were cured or completed treatment, were defaulters, or were lost to follow up.

*Case-fatality rate*: the percentage of patients who died during TB treatment out of all patients who were cured or completed treatment or were defaulters.

*Potentially unsatisfactory outcome*: the percentage of patients who interrupted treatment, were lost to follow up, or failed treatment out of all detected patients.

In accordance with guidelines of the Council for International Organizations of Medical Sciences (CIOMS, Geneva, 1991), and with recommendations of the Spanish Epidemiology Association regarding review of ethical aspects of epidemiological studies, the present study was submitted for evaluation to the Research Ethical Committee of the Teknon Medical Center, Barcelona and was approved on February 24^th^, 2006. All records with patients were identified were confidential and handled in accordance with the Spanish Data Protection Law 15/December13^th^, 1999 (Protección de Datos de Carácter Personal). Principles of the Helsinki Declaration were followed at all times. Each patient had an informed consent card read aloud to them.

### Statistical analysis

A descriptive study was carried out of the qualitative and quantitative variables collected in order to characterise the study population. Frequency distributions and medians for quantitative variables were calculated. Proportions were compared between groups using χ^2 ^tests, and when pertinent, the two-sided Fisher test. Quantitative variables were compared using Student's t-test or its non-parametric equivalent, the Mann-Whitney U-test, when assumptions of normality and homogeneity of variances were not met. Measures of association were calculated using *odds ratios *(OR) along with their 95% confidence intervals (CI). The analysis of factors associated with poor adherence treatment defaulting (comparing: cured plus treatment completed *vs. *defaulters plus lost to follow-up) and of fatality (comparing: died *vs. *the rest of patients) were analysed using logistic regression (stepwise method) including in the model the variables associated at the univariate level with a p-value < 0.15. A p-value of under 0.05 was considered significant. The test of Hosmer and Lemeshow was used to check the goodness-of-fit of the models. Analyses were conducted using the SPSS statistical package, version 13.0 (SPSS Inc, Chicago, IL, USA).

## Results

The number of patients included initially was 1500, however 10 (0.6%) had to be excluded for not meeting any inclusion criteria, so the final number of patients analysed was 1490 (table 2). The majority were males, aged >31 years old, native, occupationally active, lived with their families, diagnosed via emergency services, had pulmonary TB, either smokers or ex-smokers, initially treated with three drugs, and who understood well the implications of having TB and its treatment. They were also characterised by low frequencies of HIV infection, IDU, alcoholism, previous TB treatment, low levels of drug resistances, toxicities to drugs, and of treatment via DOT. Table 2 presents the final outcomes of therapy, where it may be noted that 89.4% of cases were cured or completed treatment. It is estimated that 1.8% defaulted, and 4.4% of cases were lost to follow-up and probably did not complete treatment.

**Table 2:** Distribution of patients in terms of study variables.

Variables		N (%)
SEX	Men	920 (63.4)
	Women	532 (36.6)

COUNTRY	Immigrant	442(29.7)
	Native	1048(70.3)

AGE	18-30	497 (33.4)
	31-50	596 (40.0)
	>50	397 (26.6)

OCCUPATIONAL STATUS	Active	901 (60.5)
	Disabled	68 (4.5)
	Unemployed	257 (17.2)
	Retired	220 (14.8)
	Unknown	44 (3.0)

LIVING ARRANGEMENTS	Alone	157 (10.5)
	Confined institutions	54 (3.6)
	Shared accommodation	189 (12.7)
	Family	1062 (71.3)
	Unknown	28 (1.9)

SOURCE	Hospital emergency department	682 (45.8)
	Primary Health Care	268 (18.0)
	Specialist	210 (14.1)
	Unknown	330 (22.1)

HIV-infected	No	1060 (71.2)
	Yes	66 (4.4)
	Unknown	364 (24.4)

INTRAVENOUS DRUG USERS	No	872 (58.5)
	Yes	21 (1.4)
	Unknown	597 (40.1)

LOCALIZATION	Pulmonary	1249 (83.8)
	Extrapulmonary	159 (10.7)
	Mixed	67 (4.5)
	Unknown	15 (1.0)

ALCOHOL USE	No	1064 (71.4)
	Yes	375 (25.2)
	Unknown	51 (3.4)

SMOKING	Non-smoker	676 (45.4)
	Smoker or ex smoker	797 (53.5)
	Unknown	17 (1.1)

INITIAL THERAPY	3 Drugs	770 (51.7)
	4 Drugs	649 (43.5)
	Unknown	71 (4.8)

DRUG RESISTANCE*	No	864 (80.1)
	Yes	85 (7.9)
	Unknown	129 (12.0)

TOXICITY	Yes	74 (5.0)
	No	1416 (95.0)

PREVIOUS TREATMENT	Yes	131 (9.0)

	No	1320 (91.0)

DIRECTLY OBSERVED TREATMENT	No	1338 (89.8)
	Yes	152 (10.2)

TREATMENT COMPREHENSION	Easy	1266 (85.0)
	Difficult	139 (9.3)
	Unknown	85 (5.7)

FINAL OUTCOME	Cured	792 (53.2)
	Completed treatment	540 (36.2)
	Failure	2 (0.1)
	Transfer out	33 (2.2)
	Default	27 (1.8)
	Death	27 (1.8)
	Lost to follow up	65 (4.4)
	Other	4 (0.3)

*Only patients with positive culture

According to these data, the outcome was 'successful' in 89.4% of patients considering all cases and 83.1% considering only smear-positive cases. Completion was 93.5% considering all TB cases and 92.4% considering only smear-positive cases. Among the immigrants, these percentages were 87.8 and 88.3, respectively. The outcome of 'potentially unsatisfactory' accounted for 6.3% of all cases and 7.4% for smear-positive cases.

The analysis of factors possibly associated to poor adherence are presented in table 3. As presented in the table, at the univariate level poorer adherence was observed for men, immigrants, younger patients, those not retired, those not living with their family, HIV-infected patients, previously treated subjects, those who had difficulty understanding the treatment, those diagnosed via emergency services, and IDU patients, whereas being in DOT had no influence. Multivariate analysis confirmed the influence of being an immigrant, living alone, being residents of confined institutions, previous TB treatment, having difficulty understanding the treatment, and being IDU.

**Table 3:** Analysis of factors associated with poor adherence to antituberculosis treatment.

			UNIVARIATE ANALYSIS (p ≤ 0.05)			MULTIVARIATE ANALYSIS (p ≤ 0.05)		
**Variables**		**N (%defaulters)**	**p-value**	**OR**	**95%CI**	**p-value**	**OR**	**95%CI**

**SEX**	**Men**	880 (7.5)	**0.015**	1.87	1.13 - 3.09			
	**Women**	506 (4.2)		1				

**COUNTRY**	**Immigrant**	420 (12.1)	**< 0.001**	3.24	2.11 - 4.98	**0.031**	2.03	1.06 - 3.88
	**Native**	1004 (4.1)		1			1	

**AGE**	**18-30**	479 (8.1)	**0.019**	2.08	1.12 - 3.83			
	**31-50**	578 (6.6)	0.109	1.65	0.89 - 3.04			
	**> 50**	367 (4.1)		1				

**OCCUPATIONAL STATUS**	**Active**	876 (6.1)	**0.028**	3.17	1.13 - 8.86			
	**Disabled**	61 (8.2)	**0.031**	4.39	1.14 - 16.92			
	**Unemployed**	244 (11.1)	**0.001**	6.12	2.10 - 17.82			
	**Retired**	201 (2.0)		1				

**LIVING ARRANGEMENTS**	**Alone**	145 (10.3)	**< 0.001**	3.27	1.74 - 6.16	**0.037**	2.35	1.05 - 5.26
	**Confined institutions**	45 (24.4)	**< 0.001**	9.18	4.30 - 19.62	**0.002**	4.79	1.74 - 13.14
	**Sharing accom.**	180 (12.2)	**< 0.001**	3.95	2.26 - 6.91	0,263	1,59	0,70-3,62
	**Family**	1029 (3.4)		1			1	

**HIV-infected**	**Yes**	60 (13.3)	**0.029**	2.40	1.09 - 5.29			
	**Unknown**	349 (6.6)	0.697	1.10	0.67 - 1.81			
	**No**	1015 (6.0)		1				

**PREVIOUS TREATMENT COMPREHENSION**	**Yes**	121 (17.4)	**< 0.001**	3.75	2.20 - 6.30	**0.009**	2.80	1.29 - 6.08
	**No**	1264 (5.3)		1			1	
	**Difficult**	122 (11.5)	**0.001**	3.60	1.91 - 6.79	**0.003**	2.93	1.44 - 5.98
	**Easy**	1238 (3.5)		1			1	

**SOURCE**	**Emergencies**	646 (9.0)	**0.006**	2.76	1.34 - 5.66			
	**Primary Care**	261 (3.4)		1				
	**Specialist**	203 (4.4)	0.587	1.29	0.50 - 3.33			
	**Other**	314 (5.1)	0.338	1.50	0.65 - 3.46			

**INTRAVENOUS DRUG USERS**	**No**	830 (4.5)		1			1	
	**Yes**	19 (21.1)	**0.003**	5.71	1.80 - 18.07	**0.001**	9.51	2.70 - 33.47
	**Unknown**	575 (8.9)	**0.001**	2.08	1.34 - 3.23	**0.027**	2.00	1.08 - 3.72

**DIRECTLY OBSERVED TREATMENT**	**Yes**	140 (7.1)	0.730	1.12	0.57 - 2.22			
	**No**	1284 (6.4)		1				

Also had no influence at univariate level: resistance, alcohol, smoking, radiology, and localization.

CI: Confidence Interval

OR: Odds ratio

The *case-fatality *was 1.8%. The analysis of factors associated with fatality is presented in table 4. Variables having an influence at the univariate level were: immigrant, disabled or retired, residents of confined institutions or incarcerated, HIV-infected, IDU, no radiological improvement, and being in DOT. Multivariate analysis confirmed the influence of being aged over 50, being retired, being HIV-infected, having comprehension difficulties, being IDU, and having been treated under DOT.

**Table 4:** Analysis of factors associated with dying during the expected treatment period among patients with tuberculosis.

			UNIVARIATE ANALYSIS (p ≤ 0.05)			MULTIVARIATE ANALYSIS (p ≤ 0.05)		
**Variables**		**N (%deaths)**	**p-value**	**OR**	**95%CI**	**p-value**	**OR**	**95%CI**

**AGE**	**18-30**	497 (0.2)		1				
	**31-50**	596 (0.8)	0.191	4.19	0.48 - 36.03			
	**> 50**	397 (5.3)	**0.001**	27.70	3.71 - 206.86	**0.039**	10.88	1.12 - 105.01

**SEX**	**Men**	920 (1.8)	0.965	0.98	0.44 - 2.16			
	**Women**	532 (1.9)		1				

**COUNTRY**	**Immigrant**	442 (0.9)	**0.099**	0.40	0.14 - 1.18			
	**Native**	1048 (2.2)		1				

**OCCUPATIONAL STATUS**	**Active**	901 (0.4)		1				
	**Disabled**	68 (5.9)	**< 0.001**	14.01	3.42 - 57.34			
	**Unemployed**	257 (0.8)	0.516	1.75	0.32 - 9.65			
	**Retired**	220 (6.8)	**< 0.001**	16.40	5.39 - 49.95	**0.012**	12.26	1.74 - 86.04

**LIVING ARRANGEMENTS**	**Alone**	157 (2.5)	0.399	1.60	0.53 - 4.83			
	**Confined institutions**	54 (5.6)	**0.045**	3.61	1.02 - 12.73			
	**Sharing accom.**	189 (0.5)	0.279	0.32	0.04 - 2.47			
	**Family**	1062 (1.6)		1				

**HIV-INFECTED**	**Yes**	66 (7.6)	**0.001**	5.71	2.00 - 16.22	**0.018**	9.93	1.48 - 66.34
	**Unknown**	364 (1.9)	0.499	1.36	0.55 - 3.37			
	**No**	1060 (1.4)		1				

**PREVIOUS TREATMENT**	**Yes**	131 (3.8)	0.092	2.34	0.87 - 6.28			
	**No**	1320 (1.7)		1				

**COMPREHENSION**	**Difficult**	139 (5.0)	**< 0.001**	7.40	2.71 - 20.21	**0.020**	4.07	1.24 - 13.29
	**Easy**	1266 (0.7)		1				

**ALCOHOL USE**	**Yes**	375 (2.4)		1				
	**No**	1064 (1.5)	0.258	0.621				
					0.27 - 1.41			

**INTRAVENOUS DRUG USERS**	**No**	872 (2.2)						
	**Yes**	21 (9.5)	**0.046**	4.72	1.02 - 21.74	**0.006**	23.59	2.46 - 225.99
	**Unknown**	597 (1.0)		1				

**RADIOLOGICAL EVOLUTION**	**Improvement**	982 (0.6)		1				
	**Stable/progression**	294 (3.1)	**0.002**	5.13	1.81 - 14.55			
	**Unknown**	214 (5.6)	**< 0.001**	9.66	3.58 - 26.04			

**DIRECTLY OBSERVED TREATMENT**	**Yes**	152 (3.9)	**0.044**	2.57	1.02 - 6.48	**0.038**	3.54	1.07 - 11.77
	**No**	1338 (1.6)		1				

Also had no influence at univariate level: resistance, smoking, and localization.

CI: Confidence Interval

OR: Odds ratio

## Discussion

In the present study, the completion outcome was 93.5% and the treatment success outcome was 89.4%, better percentages than observed in the previous study conducted by our group [6]. A study in England, Wales and Northern Ireland found a treatment success of 79% when calculated for cases in which outcome information was reported and 62% for all cases[8]. The treatment completion outcome published by the Barcelona Tuberculosis Control Program was 95.9%[9]. However, according to several studies, antituberculosis therapy adherence percentages are variable: USA[10] (91.2%); San Francisco[11] (88.6%); Norway[12] (83%); Europe[13] (69%) although the way in which theses percentages are calculated could have some influence.

In our opinion, completion outcome is a better indicator of adherence than successful outcome because is not influenced by the number of deaths (sometimes related to old patients or co morbidities but not to the quality of the Program). It is therefore essential to unify definition criteria: even though there is agreement over how to calculate the successful outcome, different methodologies are employed in calculating completion, making comparisons difficult. We consider that the ideal formula for calculating completion outcomes is that used in the present study (percentage of patients who were cured or completed treatment out of all patients who were cured or completed treatment, were defaulters, or were lost to follow-up). Furthermore, we believe it would be important to add the category 'lost to follow-up' to the European definition when it is known that the patient disappeared and no additional information is available, only considering as 'transfer out' a patient who moves to another town or changes to another health centre and whose follow-up is performed by some other physician not collaborating in the study.

Several risk factors of poor adherence have already been identified in other studies (residents of confined institutions, incarcerated, IDU, previous antituberculosis treatment, HIV-infected and immigrant) [8,14]. In our earlier study[6], the variables found to be associated were IDU and immigration while sex, age, and residents of confined institutions, incarceration, DOT or hospitalisation were not associated. In the present study, IDU and immigrant status continue to be associated, and we have also detected the influence of living alone, being residents of confined institutions, having difficulty understanding the treatment, and having previously undergone antiTB treatment. Sex, age group, occupational status, HIV status and having been diagnosed via emergency department had no influence. It is worth stressing the importance of not living with a family and the initial assessment made by the clinician in relation to the ease with which the patient comprehends the treatment. Many of those having difficulty understanding the treatment were immigrants, although some were native patients.

The case-fatality rate is low compared with other studies[15] due to the fact that in our study one of the criteria for exclusion was non-applicability of standard treatment for whatever reason (known resistances, various types of co morbidity), and also due to the fact that the frequency of HIV-infected patients with neoplasms or of advanced age was relatively low. In an European study, it was observed that advancing age and resistance to isoniazid and rifampicin were the strongest determinants of death, while male sex, European origin, pulmonary site of disease and previous anti-TB treatment were weaker predictors[16]. In an inner-city cohort, underlying illnesses such as diabetes mellitus, renal failure, chronic obstructive pulmonary disease, and HIV infection were predictors of death[17]. In Mexico, predictors of death included delays in treatment after onset of the disease and low adherence of patients to the treatment regime[18].

In our first study, the variables found to be predictive of fatality were alcoholism, HIV infection and age >64 years, whereas sex, IDU, residents of confined institutions, DOT and hospitalisation were not. In the present study, the influence of HIV and of retirement (a "proxy" of older age) is confirmed, and in addition we identify being aged over 50, being IDU, difficulties in comprehending the treatment, and being treated under DOT. In contrast, sex, immigrant status, sharing accommodation, previous antituberculosis treatment, radiological evolution, and alcoholism had no influence. When the two studies are compared, the distribution by sex, age-group and other variables are fairly similar, but the percentage of immigrants now is steadily increasing, as in other countries of Europe[19]. In relation to DOT, in the current study only 9.3% of patients were under this treatment, and in general doctors prioritise DOT for more complicated patients. In any case, it should be emphasised from analysing the predictor variables in the present study that the variable of understanding the treatment is very important not only for adherence, but also for fatality. Therefore, patients in whom the clinician observes this difficulty should be candidates for DOT and for closer monitoring in general.

In regard to the type of therapy applied, it was observed that, in line with Spanish recommendations during these years and given the low rates of primary resistance to isoniazid, the majority of native patients had received treatment with three drugs (fixed dose combinations of rifampicin, isoniazid and pyrazinamide) whereas foreign-born patients (with a higher proportion of resistance to isoniazid) were recommended to take four drugs[20], i.e. adding ethambutol. It has recently been observed that there is a progressive rise in resistances[21,22] and that this is particularly the case in the immigrant population, and hence the use of four drugs has been recommended in the treatment of incident TB patients[23], in line with both USA[24] and UK[25] guidelines.

It should be noted that the present study was carried out by a scientific society of pneumologists, and that a considerable number of collaborators contributed an extensive cohort of patients. Follow-up of cases was exhaustive, although they cannot be extrapolated to all TB patients in Spain since the study involved physicians particularly motivated by this disease. It is therefore possible that percentages of defaulting and of fatality among TB cases in Spain would be somewhat higher in general. Another limitation of this study is that patients with TB drug resistance were not included because they can have prior history of abandonment of TB treatments.

In summary, the percentage of cases coming from foreign countries is greater than recorded previously[6]. Being an immigrant, living alone, being residents of confined institutions, having a history of antiTB treatment, having difficulty in understanding the treatment, and being IDU are all factors associated with poor adherence. Death was associated with patients who were: over the age of 50, retired, HIV-infected, IDU, having difficulty understanding treatment, and being treated according to DOT (explainable since it is applied above all in the most difficult patients[26]). Therefore, to improve adherence, special care should be taken to treat patients with social problems (DOT at home, methadone programs even in prisons, admission to TB DOT centres) [27]. To reduce fatality, earlier suspicion, diagnosis, and treatment are necessary, particularly among the elderly and those patients with comorbidity or immunodepression. Community health worker intervention[28] and closer monitoring is necessary for patients in whom the physician perceives any difficulty in understanding the treatment (whether immigrants or native); this would lead not only to improved adherence, but also to better survival among these TB patients.

## Conclusion

It is important that every city, region or country studies adherence to TB treatment and its predictive factors. In our case, this study was performed by a national scientific society of pneumology and these results can help to improve the control of TB patients in our country, and in others.

## Competing interests

The authors declare that they have no competing interests.

## Authors' contributions

All authors read and approved the final manuscript. Specifically each author made the following contributions:

JAC, JRM, JC, RV, RB and JMG designed overall synthesis the study.

JAC and TR coordinated the research.

TR supervised data collection and MC analysed and interpreted the findings.

The Working Group on Completion of Tuberculosis Treatment in Spain collection the cases and reviewed the paper.
